# Reflexiones sobre innovación en salud pública: avances y retos en Colombia

**DOI:** 10.7705/biomedica.6826

**Published:** 2022-12-01

**Authors:** Gabriel Parra-Henao

**Affiliations:** 1 Instituto Nacional de Salud, Subdirección de Innovación en Salud Pública, Dirección de Investigación en Salud Pública, Bogotá, D.C., Colombia

La innovación técnico-científica es esencial para potenciar el desarrollo social y económico de un país ^1^. Tanto en el sector público como en el privado, la innovación es una fuente constante de crecimiento, ahorro de costos y mejoras en la calidad de los servicios que se prestan. Es por esto que existe un consenso mundial sobre la necesidad de potenciar los procesos de creatividad e innovación en las diferentes instituciones, partiendo de las exigencias y necesidades de las nuevas realidades ^2^ y abarcando la aceptación del riesgo, la incertidumbre y la posibilidad de fracaso; todos estos, inherentes al proceso de innovación para la creación de nuevos bienes y servicios gubernamentales, mejoras en la calidad de los bienes y servicios existentes y en la gestión de los procesos del gobierno ^3^.

El concepto de innovación en salud abarca una serie de nuevas estrategias, perspectivas, comportamientos y formas de trabajo orientadas a la solución de los diferentes problemas en salud, incluyendo, no solo tecnologías sino también mejores sistemas y políticas en salud ^4^. Según Garney, *et al.*^5^, la innovación en salud se enmarca en un proceso cíclico de retroalimentación, el cual parte de una condición existente bajo la cual se presenta una insatisfacción con las circunstancias actuales, impulsando el deseo de cambio. Para que estas innovaciones se desarrollen, se requiere de varios aspectos. Por un lado, son necesarios un clima y un contexto, es decir, unas normas sociales y culturales que las limiten o las impulsen, tales como subvenciones, apoyos económicos y restricciones. Además, son necesarios los espacios, esto es, la aprobación por parte de las organizaciones, la presencia de incentivos, recursos y habilidades para el desarrollo de la innovación. Igualmente, son imprescindibles los procesos, los cuales hacen referencia a la iteración de reflexión a partir del error, empleando el pensamiento sistémico y de diseño; y las asociaciones, que son las personas o grupos de trabajo con capacidades y prácticas para la innovación.

Este marco planteado puede ser extendido a diferentes temáticas en salud pública, donde toda innovación parte de un *status quo* y de una participación activa de las agencias o entidades gubernamentales que aportan a la generación de lineamientos y políticas públicas. En cuanto al análisis holístico y el pensamiento de diseño, es necesaria la identificación de relaciones y patrones en los problemas de salud pública, evitar el enfoque de búsqueda de una causalidad lineal y tener en cuenta las interacciones de diferentes factores, siempre partiendo de las necesidades de la población objetivo, sus comentarios y apreciaciones para satisfacer directamente a los pacientes y a la comunidad en general a la cual está dirigida la innovación. Finalmente, cabe destacar que el desarrollo de las capacidades de innovación solo se produce con la experiencia y que las personas sean flexibles en su pensamiento; por tanto, se debe evitar ingresar a un proyecto con un resultado predefinido en mente para luego alcanzarlo ^5^.

En Colombia, se han dado avances en ciencia, tecnología e innovación. En el 2021 se actualizó la política nacional de ciencia, tecnología e innovación y se emitió el documento CONPES 4069 ^6^, el cual busca convertirnos en uno de los tres países líderes de Latinoamérica y lograr una inversión nacional del 1 % del PIB en investigación y desarrollo, lo cual impactaría positivamente al país en lo social, lo económico y lo ambiental. El documento incluye el incremento de las capacidades de las instituciones generadoras del conocimiento y de las entidades de soporte a través de una estrategia para fortalecer el ecosistema científico del país que incluye:

“[...] la formulación e implementación del plan estratégico para la integración de Institutos y centros públicos de investigación, que, manteniendo su identidad y autonomía, les permita compartir capacidades e infraestructuras para el desarrollo de proyectos de CTI [...]” (Consejo Nacional de Política Económica y Social, 2021) ^6^.

En el marco de este CONPES, el ecosistema de ciencia, tecnología e innovación en Colombia cuenta con un amplio escenario de posibilidades de desarrollo de innovación en salud. De acuerdo con las cifras de la última medición de grupos de investigación de Minciencias ^7^, de los 6.160 grupos de investigación reconocidos en Colombia, el 17 % pertenece a las ciencias médicas y de la salud. Estos pertenecen a las universidades y centros e institutos, mixtos o privados, que desarrollan investigación e innovación en el área. En cuanto al sector público, en el área de salud, cinco instituciones generadoras del conocimiento cuentan con capacidades, conocimientos, personal y líneas de investigación y equipos para aportar a la generación de bienes públicos en el área de la salud: Instituto Nacional de Cancerología, Centro Dermatológico Federico Lleras Acosta, Instituto de Evaluación de Tecnologías en Salud, Instituto Nacional de Salud y el Instituto Nacional de Medicina y Ciencias Forenses ^8^.

No obstante, las ideas valiosas pueden provenir de adentro de la entidad o instituciones generadoras del conocimiento, o fuera de esta. Actualmente, una de las estrategias de innovación que ha cobrado fuerza es la innovación abierta, en la cual, mediante la colaboración, se reducen riesgos y se aprovechan conocimientos, capacidades y experiencias que pueden aportar actores externos involucrados en la solución de un problema a través de un clúster de innovación, el cual reúne una serie de partes interesadas que comparten desafíos y proponen iniciativas e intervenciones para solucionarlo ^9^. La pandemia, por ejemplo, ha sido un desafío para muchos sistemas de salud pública. La innovación en salud y la articulación de diferentes actores son una forma eficiente y efectiva de dar respuesta donde se requiere la capacidad de asociación para concebir ideas disruptivas, crear estrategias y políticas que permitan la incorporación de soluciones a corto, mediano y largo plazo para los diferentes problemas que se avecinan en esta época de postpandemia ^10^.

Además, la evolución y los constantes cambios de las nuevas tecnologias como el internet de las cosas, la nube, el *Big Data*, las *smart cities*, la inteligencia artificial, la impresión 3D, la medicina personalizada, el *blockchain* y los robots, entre otras, siguen modelos basados en plataformas tecnológicas, la cual es otra estrategia que permite la generación de innovaciones a través de procesos de colaboración con socios o partes interesadas de un ecosistema; esto para promover estilos de vida saludables, prevención primaria y secundaria y el diagnóstico temprano, donde se involucran diferentes componentes que permiten crear modelos de asociación técnico-científico y que complementen las capacidades resolutivas ^11^.

Por otra parte, otra de las estrategias aplicadas a la salud pública es la innovación social. Esta permite encontrar nuevas y efectivas soluciones a problemas de salud generando impactos sociales tangibles. La innovación social ha ganado fuerza a nivel gubernamental debido a experiencias anteriores que resultaron en la ineficiencia de algunas políticas públicas, las cuales han buscado alcanzar metas que distan ampliamente de la realidad de las comunidades. Esta estrategia demanda la desaparición de la inmediatez, la reducción de la certidumbre y la necesidad de continuidad de las políticas. Asimismo, involucra a la sociedad para resolver problemas a través de metodologías abiertas que van desde lo digital hasta lo ancestral y que estrechan vínculos entre el gobierno, la sociedad civil, las empresas privadas y las organizaciones no gubernamentales ^12^.

Como se puede evidenciar, la innovación en salud pública en el país puede apalancarse con estrategias que dan cabida a la participación de múltiples actores en búsqueda del beneficio de las comunidades. De esta manera, instituciones como el Instituto Nacional de Salud han creado dependencias como la Subdirección de Innovación en Salud Pública para apoyar este aspecto importante para el avance del ecosistema de salud del país. Asimismo, desde el Ministerio de Ciencia, Tecnología e Innovación del país se han impulsado estrategias innovadoras propuestas desde universidades y centros de investigación para afrontar la reciente pandemia por COVID-19. Todas estas estrategias, sumadas a otras iniciativas en innovación en salud pública de diferentes entidades, nos permite ver con optimismo el avance del ecosistema de innovación en el país. El reto futuro es continuar apoyando las estrategias de innovación en salud pública y asegurar su financiamiento a través de diferentes mecanismos del orden nacional, departamental, municipal y del sector privado.
